# Discrepancy between experience and importance of recovery components in the symptomatic and recovery perceptions of people with severe mental disorders

**DOI:** 10.1186/s12888-021-03287-y

**Published:** 2021-05-31

**Authors:** Patricia Penas, Jose-Juan Uriarte, Susana Gorbeña, Mike Slade, María-Concepción Moreno-Calvete, Ioseba Iraurgi

**Affiliations:** 1 University of Deusto, Avda. de las Universidades 24, 48007 Bilbao, Spain; 2 Biocruces Bizkaia Health Research Institute. Basque Health Service, Bizkaia Mental Health Network, Plaza de Cruces 12, 48903 Barakaldo, Bizkaia Spain; 3 School of Health Sciences, Institute of Mental Health, University of Nottingham, Nottingham, UK

**Keywords:** Personal recovery, Orientation of mental health services, Severe mental disorder, Clinical recovery, Internalized stigma

## Abstract

**Background:**

Personal recovery has become an increasingly important approach in the care of people with severe mental disorders and consequently in the orientation of mental health services. The objective of this study was to assess the personal recovery process in people using mental health services, and to clarify the role of variables such as symptomatology, self-stigma, sociodemographic and treatment.

**Methods:**

Standardised measures of personal recovery process, clinical recovery, and internalized stigma were completed by a sample of 312 participants in a Severe Mental Disorder program.

**Results:**

Users valued most the recovery elements of: improving general health and wellness; having professionals who care; hope; and sense of meaning in life. Significant discrepancies between perceived experience and relative importance assigned to each of the components of the REE were observed. Regression modeling (χ^2^ = 6.72, *p* = .394; GFI = .99, SRMR = .03) identified how positive discrepancies were associated with a higher presence of recovery markers (β = .12, *p* = .05), which in turn were negatively related to the derived symptomatology index (β = −.33, *p* < .001). Furthermore, the relationship between clinical and personal recovery was mediated by internalized stigma.

**Conclusions:**

An improvement in psychiatric services should be focused on recovery aspects that have the greatest discrepancy between importance and experience, in particular social roles, basic needs and hope. Personal and clinical recovery are correlated, but the relationship between them is mediated by internalized stigma, indicating the need for clinical interventions to target self-stigma.

## Background

The concept of recovery in people with severe mental disorders (SMD) has undergone profound changes over the past few decades, transforming mental health systems internationally [1, 2]. Supporting personal recovery involves enabling individuals to lead meaningful lives with values, purposes, and relationships that are meaningful to them, despite the limitations caused by the illness [3]. Therefore, the active role of service users is an essential aspect in the recovery process [4], and as a consequence it is important to take into account the experience of the users themselves when evaluating both their process of recovery and the assistance they are receiving in mental health services [5].

Although the conceptualization of personal recovery has been challenging because of the individuality and subjectivity of the process, the CHIME conceptual framework has gained wide international acceptance and validity [6, 7]. This conceptual framework highlights five recovery processes: Connectedness, Hope and optimism, Identity, Meaning and purpose and Empowerment, which in turn are divided into several sub-categories [8]. Thus, the importance that service users assign to the different components of personal recovery may be of interest for knowing which of these elements are the most valued by users. If the experience of how services support the different components is evaluated, improvements could be made in the different areas, focusing on those that can be considered the most deficient. In this sense, it is important that the elements considered significant for recovery process are addressed by services. If there is a large gap between what a person feels important to his/her recovery and the experience of the extent to which this element is promoted by services, this may make recovery less likely. A moderate distance between the importance attributed and the subjective experience of those elements may facilitate individuals to act and take responsibility for their personal recovery journey. This fact could be related with cognitive dissonance theory, which indicates that consistency between beliefs and perception facilitates action, and inconsistency generates discomfort and less probability of engaging in action [9, 10].

In some cases, services inconsistently address recovery principles as they continue to focus on the traditional medical model, and do not efficiently cover the needs that users consider important for their recovery [11, 12]. In this sense, it cannot be forgotten that personal recovery involves the active role of the person, a journey that should be supported and promoted by mental health services. Therefore, the user’s own voice and perspective regarding their individual process, recovery facilitators and barriers should be considered and included [13].

Some studies suggest that the previously mentioned traditional clinical recovery, which refers to remission or reduction of symptoms, is not incompatible with the more current perspective of personal recovery, but both processes are connected and complementary [14, 15]. However, Roe and his colleagues [16] found that the relationship between a global index of symptomatology and personal recovery was not significant, suggesting that the two types of recovery do not have a direct relationship. A recent meta-analysis, found that personal and clinical recovery are distinct outcomes [17], and Van Weeghel and colleagues [18] pointed out that both types of recovery differ in their conceptualization. Promoting both types of recovery interventions is a complex challenge, as the objectives promoted by each type of recovery are different [18].

Additionally, the stigma that these individuals experienced as a consequence of the illness, is an important barrier in the recovery process. After the diagnosis of the disorder, it is common for people to notice how their social relationships diminish, or how they suffer a devaluation of their opinions or skills [19]. When these attitudes or prejudices are internalized by people, self-stigma or internalized stigma arises [20], which in many cases leads to a reduction in self-esteem, self-efficacy, empowerment and hope, and also an increase in symptoms and reductions in treatment adherence or other activities that involve relating to others [21]. Since self-stigma has a negative influence on some important aspects of the recovery process, and makes it difficult to achieve personal functioning, hope or full participation in the community [22, 23], different specific interventions have been developed to reduce self-stigma [24, 25]. Considering the importance given to internalized stigma and its relationship with dimensions related to personal recovery [25] and symptoms [21], it would be useful to clarify the role that this variable might play when both perspectives of recovery are taken into account.

The aim of this study was to evaluate the process of personal recovery in people using mental health services, and to explore variables that may be interacting or mediating in the process of personal recovery.

Objective 1 was to assess the relative importance that users assign to the different elements of personal recovery, as well as the subjective experience that they perceive in relation to how these elements are addressed in the services. This allows a differential score to be obtained as a proxy measure of the discrepancy between the experience perceived by each user with respect to the elements of recovery and their assessment of these elements.

Objective 2 was to explore the association between personal recovery variables, clinical variables and socio-demographic variables, in order to assess the predictive effect of the sociodemographic and treatment variables, self-stigma and the discrepancy generated about the recovery performance in the services, on the personal and clinical recovery variables.

Objective 3 was to estimate a model capturing the role of the different variables in the process of recovery from a severe mental disorder.

## Method

### Sample

Based on the census (N = 1949) of active clinical cases attended by the Severe Mental Disorder Programme (SMDP) of the Bizkaia Mental Health Network (Spain), a random stratified selection strategy according to sex, age and type of mental health service attended (Mental Health Centre, Day Hospital, Community Assertive Treatment, Psychiatric Hospital) was used. A sample size of 300 participants was considered acceptable, with an associated estimation error of 5.2% for a 95% confidence level.

Inclusion criteria were: being at least 18 years old and a user of the SMDP. Exclusion criteria were: absence of informed consent, language or communication problems, and presence of significant clinical symptoms.

### Participants

A total of 312 SMDP users participated, reducing the estimation error to 5.1%.The most common diagnosis was schizophrenia (56.1%), then bipolar (12%) and schizoaffective disorders (8.9%), and the remainder were classified withother disorders such as depression or personality disorders, with an average of 17.37 (SD = 8.70) years in treatment. 189 participants were males and 123 females with an average age of 48.75 (SD = 11.00) years old. 62.2% (*n* = 194) of the sample were treated in outpatient mental health facilities, 24% (*n* = 75) in day-care hospitals, 7.1% (*n* = 22) in Assertive Community Treatment and 6.7% in hospital settings.

### Measures

#### Personal recovery

The *Recovery Enhancing Environment* measure (REE) [26], also known as DREEM in United Kingdom [27], was used in its Spanish version [28]. It is an instrument composed by four independent sections: 1) importance of 24 recovery elements, related to recovery CHIME conceptual framework [8, 29], 2) experience of how those 24 elements are addressed in the services, 3) organizational climate and 4) recovery markers to assess the personal process of recovery, which has been used independently in some studies as Recovery Markers Questionnaire [30]. In this research the subscale has been used to assess subjective personal recovery. The items are scored using a five-point Likert scale, in which higher scores are indicating more positive results.

#### Clinical recovery

The *Clinical Global Impression* scale (CGI) [31] was used in its Spanish version [32] to evaluate severity and the clinical progress of the service users. The clinician completed two subscales each ranging from 0 (not assessed) to 7 (most severely ill patients/very much worse): severity of psychopathology and global improvement or change.

The *Global Assessment of Functioning* scale (GAF) [33] – Spanish version [34] was used to measure global functioning. The clinician ranks psychological, social and work activities on a continuum of mental health-illness, from 1 (severely impaired) to 100 (maximum functionality).

The *Health of the Nation Outcome Scale* (HoNOS) [35, 36] was used to measure the severity of the disorder. HoNOS consists of 12 items with four subdimensions: behavioural problems, impairment, clinical problems and social problems. Each item is scored on a five-point Likert scale from 0 (no problem) to 4 (severe problem).

Health related quality of life was assessed using the analogue visual scale (EVA) implemented through the *EuroQol-5D* scale [37, 38]. The person indicates, using an interval from 0 (worst imaginable state) to 100 (best imaginable state) the point that best reflects his or her current overall state of health.

Using these four instruments a global index of symptomatology was created in order to assess clinical recovery in relation to reduction or remission of symptoms.

#### Assessment of self-stigma

The *Internalized Stigma of Mental Health Illness* (ISMI) [39, 40] was used to assess subjective experience of self-stigma in people with mental illness. It is a 29-item scale with each itemrated on a four-point Likert scale from 1 (totally disagree) to 4 (totally agree), and divided into five subscales: alienation, stereotype endorsement, discrimination experience, social withdrawal and stigma resistance.

### Procedure

Participants were randomly selected from a stratified sample. If the selected person did not satisfy the inclusion criteria or did not give informed consent, another user from the same stratum was selected randomly to replace them. Once participants accepted, they completed the REE instrument through an interview, and the EuroQol-5D and ISMI scales. Clinicians (psychiatrists and clinical psychologists) with clinical responsibility for the participant completed the HoNOS, CGI and GAF. Those instruments are used routinely in the evaluation protocols, and so clinicians have been trained and have experience in completing those scales in a homogeneous way. The REE interviewers were four service users with personal experience in the recovery process, who were hired to collect the data, as part of an ongoing strategy of patient empowerment. They received training on the characteristics and application of the REE and in interviewing skills. The research had the approval of the Clinical Research Ethics Committee of the Health Services of the Basque Country.

### Statistical analysis

For Objective 1, the central tendency measures, to characterise the difference between both scores the contrast test means (*Student’s t-test pairs*) and the size effect (*Cohen’s d coefficient*) were calculated. A global indicator for the discrepancies between the importance and experience of the 24 elements of the REE was obtained, called the differential score. A positive or null differential score (positive discrepancy) indicates that the experience of an element is above its importance, indicating a strength of the system that has made it possible to reach it. A negative differential score (negative discrepancy) indicate that the achievement has not been reached, and therefore, it is an area that should be supported more in the services.

For Objective 2, an indicator of clinical symptomatology was obtained from the instruments used to measure clinical variables. The scores of the HoNOS, CGI, GAF and EVA-EuroQoL instruments were subjected to an exploratory factor analysis. A single factor solution explaining 58.20% of the variance was obtained, where the standardized factor scores were used as an expression of symptomatology. The degree and significance of the associations between variables was estimated using Pearson’s correlation coefficients. The analysis and selection of the possible predictive factors of Symptomatology and Recovery were conducted through hierarchical linear regression techniques, using a step-by-step introduction process. The specification of these steps is explained in detail in the results section. The type of care service was considered among the predictive variables, so for its inclusion in the regression model the nominal variable was transformed into a set of dummy variables where the category ‘Psychiatric Hospital’ is taken as a reference for the estimation of the response in the rest of the options (‘1’ Mental Health Centre, ‘2’ Day Hospital and 3 ‘Assertive Community Treatment).

For Objective 3, a structural model was constructed to explain the relationship between the following variables: differential scores between experience and importance, self-stigma, and personal and clinical recovery. The path analysis was calculated in the EQS program considering the following adjustment indices: the chi-squared test (χ2); the Goodness for Fit Index (GFI); the Adjusted Goodness for Fit Index (AGFI); the Comparative Fit Index (CFI), which values should be >.90; and finally, Standardized Root Mean Square Residual (SRMR) and the Root Mean Square Error of Approximation (RMSEA), where values <.05 are considered adequate, with a 90% confidence interval.

## Results

Table 1 presents the mean values expressed by participants for each of the 24 elements assessed by the REE that composed the recovery construct, both in their level of experience and the importance attributed to each of them. Among the most valued components in importance (M ≈ 3.50) are ‘improving general health and wellness’, ‘professionals’ who care about’ and ‘hope’. And among the least experienced (M < 2.0) ‘intimacy and sexuality’ and ‘spirituality’, being these elements also the least appreciated in terms of importance. Also, the difference between their experience and importance is offered. Except in the case of stigma (I19), in all cases the differential score was negative, in other words the personal experience is less than the importance that the person attributed to the component. In all cases the differential score was statistically significant (*p* < .024). Of the 24 components, 20 offer appreciable effect sizes (d > .40), and six of them very high effect sizes (d > .80). Only four components have low effects (d < .35).

**Table 1:** Differences between the subjective experience and importance in 24 recovery elements

items	Experience		Importance		Differential score	T Student	df	p	r	Cohen’s d
	M	SD	M	SD						
6. Improving general health and wellness	2.96	0.76	3.49	0.62	−0.53	11.35	310	.001	.31	0.76
24. Professionals who care about	3.22	0.67	3.49	0.63	−0.27	6.87	310	.001	.45	0.41
3. Hope	2.76	0.73	3.48	0.69	−0.72	15.05	310	.001	.30	1.01
2. Sense of meaning in life	3.02	0.69	3.46	0.69	−0.44	9.38	309	.001	.30	0.64
5. Self-manage symptoms /avoid relapse	2.89	0.77	3.43	0.70	−0.54	10.24	311	.001	.20	0.73
15. Basic needs	2.58	0.82	3.41	0.72	−0.83	15.86	311	.001	.28	1.07
16. Sense of control and empowerment	2.91	0.74	3.39	0.69	−0.48	10.43	311	.001	.34	0.67
1.Positive sense of personal identity	3.08	0.69	3.38	0.71	−0.30	5.95	310	.001	.19	0.43
12. Having positive relationships	2.63	0.88	3.38	0.78	−0.75	13.19	311	.001	.27	0.90
13. Identifying and building on strengths	2.83	0.83	3.37	0.69	−0.54	10.92	309	.001	.36	0.70
22. Assistance in crisis	3.01	0.76	3.37	0.74	−0.36	8.78	308	.001	.54	0.48
8. Having rights respected and unheld	2.69	0.78	3.34	0.72	−0.65	12.63	311	.001	.26	0.87
18. Taking on/succeeding in social roles	2.08	0.98	3.34	0.71	−1.26	21.05	311	.001	.24	1.46
7. Being active and directing the recovery	2.71	0.75	3.26	0.75	−0.55	10.39	310	.001	.25	0.73
10. Being involved in meaningful activities	2.81	0.80	3.25	0.82	−0.44	8.90	309	.001	.42	0.54
11.Involved and take part in community	2.77	0.83	3.23	0.89	−0.46	8.39	310	.001	.36	0.53
20. Taking on new challenges	2.85	0.83	3.12	0.90	−0.27	5.13	310	.001	.45	0.31
14. Developing new skills	2.69	0.85	3.10	0.80	−0.41	7.41	311	.001	.31	0.50
21. Having positive role models	2.33	0.74	3.08	0.84	−0.75	14.5	310	.001	.34	0.95
4. Having up-to-date knowledge	2.73	0.81	2.98	0.88	−0.25	4.48	311	.001	.28	0.30
9. Mutual self-help/peer support	2.58	0.80	2.79	1.07	−0.21	3.63	311	.001	.44	0.22
23. Intimacy and sexuality	1.78	0.98	2.78	1.14	−1.00	13.93	308	.001	.28	0.94
19. Challenging stigma/ discrimination	2.61	0.85	2.45	1.27	0.16	−2.27	311	.024	.37	0.14
17. Spirituality	1.38	0.95	2.06	1.36	−0.68	10.25	311	.001	.53	0.56

Note. The elements are ordered according to the importance

Regarding the association between variables (Table 2), there are several statistically significant correlations (r ≥ .30; *p* < .05) in the direction that theoretically would be expected: 1) positive associations between recovery indicators (experience, importance and recovery markers), 2) a negative association of recovery markers with self-stigma (r = −.51) and with symptomatology (HoNOS r = −.36; Index of symptoms r = −.41), and 3) a positive association between stigma and symptomatic indicators. The variables age, sex and years in treatment showed low associations with recovery variables, symptoms and perceived self-stigma.

**Table 2:** Descriptive statistics and correlation matrix

	M	SD	α	1	2	3	4	5	6	7	8	9
1. Experience	2.66	0.56	.95									
2. Importance	3.18	0.47	.90	.56**								
3. Differences exp-imp	−0.52	0.49	–	.61**	−.32**							
4. Recovery markers	2.68	0.66	.93	.48**	.41**	.16**						
5. Time in treatment	17.37	8.70	–	−.02	.04	−.06	−.02					
6. Age	48.75	11.00	–	−.08	−.10	−.00	.02	.37**				
7. Gender	–	–	–	−.08	−.02	−.08	−.00	.02	.16**			
8. Internalized stigma	1.99	0.47	.92	−.15**	−.09	−.09	−.51**	.07	.05	−.07		
9. HoNOS	11.30	5.98	.70	−.11	−.02	−.11	−.36**	.05	−.09	−.05	.30**	
10. Symptoms Index	0	1.00	–	−.13*	−.05	−.10	−.41**	.08	−.04	−.04	.33**	.87**

Note. Significance level *.05 and **.001

Given the results obtained from the correlations, two hierarchical regression models were conducted to explore the variables involved in the symptomatic response (Table 3) and in the recovery markers (Table 4). The indicator that assesses discrepancy between recovery experience and importance attributed to recovery was the first input factor in both models. The second step of each regression varied depending on the response variable: recovery markers as a predictor of the symptomatology (Table 3), or symptomatology as a predictor of the recovery markers (Table 4). The next step included sex and age as sociodemographic control variables (step 3). Then the type of care service in which participants attended (step 4), formulated as a dummy variable taking as reference participants who attended a psychiatric hospital was introduced. And finally, self-stigma was included in the model (step 5).

**Table 3:** Regression model considering the Global Index of Symptomatology

	Step 1		Step 2		Step 3		Step 4		Step 5	
	β	p	β	p	β	p	β	p	β	p
Differences exp-imp	−.100	.106	−.034	.555	−.028	.620	−.011	.842	−.012	.829
Recovery Markers			−.410	.000	−.405	.000	−.405	.000	−.317	.000
Age					−.069	.259	−.024	.697	−.041	.490
Gender					−.044	.438	−.045	.420	−.031	.574
Time in treatment					.096	.114	.114	.055	.104	.077
Services										
D1							−.431	.001	−.426	.001
D2							−.246	.037	−.241	.038
D3							−.103	.211	−.090	.272
ISMI									.175	.006

Note. Dummy variables of the services (D1-CSM, D2-HD, D3-TAC)

**Table 4:** Regression model considering the Recovery Markers

	Step 1		Step 2		Step 3		Step 4		Step 5	
	β	p	β	p	β	p	β	p	β	p
Differences exp-imp	.161	.009	.121	.032	.118	.039	.140	.017	.118	.027
Index of Symptoms			−.403	.000	−.403	.000	−.426	.000	−.283	.000
Age					.027	.652	.042	.494	.078	.168
Gender					−.036	.531	−.034	.549	−.057	.272
Time in treatment					.002	.979	.018	.769	.030	.589
Services										
D1							−.266	.038	−.200	.086
D2							−.237	.049	−.189	.085
D3							−.099	.243	−.106	.168
ISMI									−.405	.000

Note. Dummy variables of the services (D1-CSM, D2-HD, D3-TAC)

Regarding the factors associated with symptomatology (Table 3), the differential score between experience and importance attributed to recovery did not show a significant effect on symptomatology, nor on its total effect (β = −.10; step 1), nor once it is controlled by remaining variables (β = −.01; step 5). However, there was an effect on recovery markers (β = −.41; step 2), which remained stable when controlling for socio-demographic and clinical variables (β = −.40; step 3 and 4), and was slightly attenuated when the self-stigma variable was introduced (β = −.32; step 5). Likewise, this last variable of self-stigma offered a statistically significant effect, although with a less strong relationship than recovery markers (β = −.17 vs β = −.32; step 5) on the expression of symptoms.

In Table 4, the second hierarchical regression model is presented, in this case with the prediction of recovery markers. For the differential score between experience and the importance attributed to recovery there was a statistically significant effect on recovery markers, although with a moderate-low effect, both in its total effect (β = .16; step 1) and when controlling for the remaining variables (β = .12; step 5). The symptomatology index had a noticeable effect on recovery markers (β = −.40; step 2), which remained stable when controlling for socio-demographic and clinical variables (β ≈ − 40; step 3 and 4), and somewhat attenuated when the self-stigma variable is introduced (β = −.28; step 5). As in the previous model, the self-stigma variable had a statistically significant and relevant effect (β = −.40; step 5) on recovery markers.

**Fig. 1:**
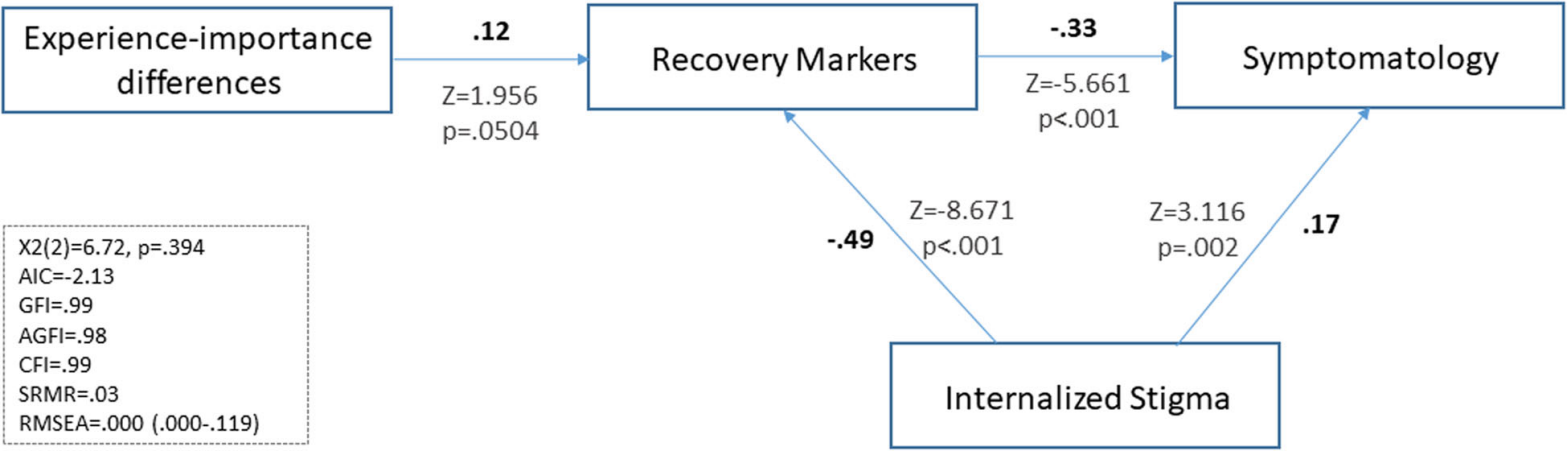
The equation model of recovery

Finally, the combination of the present results guided the configuration of the model with the relationships between variables, which is expressed in the path-analysis shown in Fig. 1. The fit indices (χ2_(2)_ = 6.72, *p* = .394, AIC = -2.13, GFI = .99, AGFI = .98, CFI = .99, SRMR = .03, RMSEA = .000 [.000–.119]) indicated an adequate model. Symptomatology was mainly explained by having low recovery indicators (β = −.33) andto a lesser extent high self-stigma scores (β = .17), which in turn had a significant influence in recovery markers (β = −.49). On the other hand, a greater discrepancy between experience and importance attributed to recovery impacted on recovery markers, although this relationship was moderate and at the limit of statistical significance (β = .12; *p* = .0504).

## Discussion

The objective of the present study was to evaluate the personal recovery process in people with severe mental disorder. First, elements associated with recovery, and the differential score resulting from the difference between the importance that the service users give to the different recovery elements and how these are managed by the care services, could be an indicator of interest in identifying different areas of work in mental health services. Second, the association between the different variables and their predictive effect in relation to personal and clinical recovery was investigated.

The components related to the improvement of health and well-being, having professionals who really care and demonstrate active listening, having hope, meaning of life, and managing and controlling symptoms, were considered by users as the most relevant elements in their recovery process. Some researchers [8, 41] found hope or feeling that life has meaning as key elements in the recovery process. However, elements that refer to the management and control of symptoms to prevent relapse, or to improve physical health and well-being, may have appeared in the literature as less salient elements [42] or as elements implicit in others [8], or even components more related to the clinical recovery model, where the indicator of improvement is associated with the reduction or remission of symptoms [14]. But in this case, users considered that being able to identify warning signs of possible relapses or knowing how to control their symptoms was important for their recovery, which is closely related with taking an active role in their recovery. In this sense, Deegan [43] indicates that recovery is carrying out a life in spite of the limitations caused by the illness, and therefore knowing how to use medication to control symptoms is also part of the recovery process.

Although these have been the five components considered most important, when assessing the discrepancy between valued and experienced elements in the services, the components with higher effect sizes were: 1) taking on, and succeeding in, normal social roles, obtaining a job, being a successful student, or securing housing and being a successful tenant; 2) having basic needs met, which means, obtaining a basic economic income, securing affordable housing and receiving necessary health care; 3) having hope; and finally, 4) having positive role models. This differential scores are interesting, not only because they provide information about the aspects that should be improved in mental health services delivery, but also because this dissonance could be affecting the users’ own personal recovery process. Therefore, those aspects or elements should be considered when proposing changes or alternatives in those services.

The components of having basic needs met and re-integrating into society are considered key elements in the process of personal recovery, being realms of life that are not only important for mental health services users, but for any individual in society. The possibilities of re-entering the job market for people with a severe mental disorder are reduced [44], which makes it difficult for them to lead their lives independently [45] and to satisfy some basic needs such as income or adequate housing. Although provision of these economic and social resources is not a direct responsibility of mental health services, it is important to consider them in order to improve aspects that can be related to greater satisfaction of these components: the improvement of information regarding the different procedures to obtain economic aid, training in search of employment, or training in social skills, among others. Nevertheless, hope has been considered in the literature as a key element in the process of mental health recovery [8, 46]. Consequently, it is necessary that mental health systems promote hope and positive attitudes for the future, with the aim that users would consider the possibility of recovering and begin able to take steps towards achieving a satisfactory life [46]. Finally, the dissonance found between importance and experience in relation to having positive role models suggests that the inclusion of mutual support or peer-support programs in services should be routinely available. Those programs involve hiring experts in recovery process to help others in less advanced stages of the process [47]. There is robust evidence that the inclusion of peer workers in mental health services is an important contributor to recovery [48]. Peer workers provide practical and emotional support to users, who increase hope and empowerment, reduce stigma, improve their active process and commitment to services, while promoting a better relationship with professionals [49, 50]. The effects of including these experienced users in mental health services not only has a positive impact in the recovery process of other patients, but also on other professionals, services that became more recovery-oriented, and on peer supporters themselves [51], as they develop a greater sense of personal, social and occupational wellness [52]. Therefore, role models in the services could be a good alternative to improve those components of hope and good examples to follow, and they would also be offering these people the possibility of being able to participate in society through the acquisition of paid employment that in turn would cover their basic needs.

In the correlational analysis between the clinical (symptomatological index) and personal (recovery markers) recovery variables, significant results suggested a negative relationship between these two variables. In other words, greater personal recovery was associated with reduced clinical symptomatology. Likewise, the internalized stigma variable correlated negatively with the recovery markers and positively with the symptomatology, which would indicate that the higher internalized stigma was associated with lower personal and clinical recovery. The component of stigma and discrimination was not perceived as an important element for the recovery of users, however, the high correlation of self-stigma with both types of recovery suggests that it is a variable that may be influencing the process. In this sense, in the study conducted by Whitley and Campbell [53] they found that although stigma and discrimination were not considered as problems normally experienced by their participants, they were pervasive issues to deal with, such as striving to behave or dress normally. These authors also associated these normalization strategies with the tools provided by recovery-oriented services or communities [53].

The results of the regression show that the sociodemographic variables of age, sex and years in treatment do not moderate the variables of recovery markers and symptoms index. However, as expected the type of service variable moderated the predictive variable of symptoms, since severity was a criterion used to select the appropriate treatment resource. Therefore, these results indicate that both recovery variables are independent of sex, age or treatment time.

Finally, the structural model, configured from regression analysis´ results, shows a model where recovery markers have a negative relationship with the symptomatology, which is a relationship mediated by internalized stigma. However, there are studies in the literature that have not found an association between the scales that assess personal recovery and those that evaluate symptomatology variables, suggesting that both types of recovery are not the same, although they suggest that they may be complementary processes [16, 54]. In this sense, Chan and his team [55] found that personal recovery was a predictor of well-being beyond clinical or functional recovery, so that people with schizophrenia are able to achieve personal recovery despite symptomatic or functional limitations. In contrast, and in line with what was found through this study, other research has also obtained associations between aspects of personal and clinical recovery [17], suggesting that the integration of both models could improve the evaluation and understanding of the process [56]. Thus, self-stigma has been associated more strongly with the personal recovery indicator than with the clinical one, which would suggest that it is a process related to the different abilities that the person develops during his/her personal recovery process, such as empowerment or hope [21]. In this regard, Vass and his team [57] conducted a longitudinal study where they found that stigma predicted both subjective and clinical recovery. Moreover, internalized stigma has been identified as an important barrier in recovery [23]. This could also be in line with illness identity model proposed by Yanos and team [58], which suggests that the internalized stigma acquired by people suffering from a severe mental illness affects their identity. In turn, this identity has an effect on the role that the people takes in their journey of recovery converting this into a more passive one and affecting it objectively as well as subjectively [58]. All this could be indicating that it is necessary to work with stigma and social prejudices that these people have internalized as a result of their social experiences, since it seems that the role of self-stigma could have an important impact in their process of recovery.

An element to highlight from the variables analyzed in this study is the role of the discrepancy indicator or differential score between the experience attributed to the different recovery components and the importance attributed to them. A positive discrepancy means that the experience is greater than the importance and, therefore, it can be assumed that the user has reached a level of satisfaction with such a component. By contrast, a negative discrepancy would indicate that there is still work to be done to satisfy the user’s progress with that recovery component, which could lead to a state of dissatisfaction and/or frustration. It was observed that a greater positive discrepancy was slightly associated with a higher score on recovery markers. In other words, achievements in the recovery process would be more present among people who have experienced more in recovery elements than the importance attributed to them. This hypothesis allows us to suggest that the REE offers a list of components to assess the discrepancy between the experience and importance attributed by a specific user and select those components that are more deficient (negative discrepancies), in order to establish a personalized planning in their recovery process and support them in their own recovery journey. Thus, identifying which components are deficient would allow us to plan specific interventions for their achievement, while reinforcing or stabilizing those that have already been achieved.

Some limitations can be identified. The first is in the type of design used. A cross-sectional study does not allow for the establishment of causality. Although regression models are used and reference is made to the term predictor and outcome variables, this is still methodological jargon. It is necessary to replicate the study by means of a longitudinal design, where the evolution and changes in the REE scores and other variables considered would be analyzed, and consequently it would be possible to assess if baseline time estimates can explain the achievements at a later point in time. For the time being, the set of relationships found in this study allow us to offer working hypotheses that may be interesting in the study of recovery processes in severe mental disorders. Clinical recovery has been assessed with instruments that are broadly used in clinical settings, but rater by the clinician responsible for the mental health service user; consequently, rated bias could have an impact on this variable. Finally, the focus of this study has been on the service contribution to recovery, and in future research a wider focus on the service user’s power to influence their own recovery is needed, along with the support they receive from their community and peers.

While a considerable effort was made to secure a sufficient number of participants to reduce sampling error, this does not guarantee that there has not been a selection bias. The exclusion/rejection rate for participation in the study was relatively significant - around 30.7% - and although there was replacement with another candidate with equivalent sample selection characteristics, it was not possible to establish whether there was equivalence in other variables of clinical interest - for example, the degree of severity of their illness - that could influence their recovery process. It is plausible that individuals in a more advanced process of recovery are more likely to agree to participate in the study, and if so the results would be biased towards this subgroup of people. The systematic application of the REE in a system of care for severe mental illness as an assessment tool in the initial diagnosis and in the periodic monitoring would provide data on the entire population of users.

To conclude, this study found that the differences between people’s perceived importance and experience of the different components of recovery, and how these are addressed in services, could influence their own personal recovery process. Also, the relationship found between personal and clinical recovery suggests that both types of recovery are interconnected, so services should promote strategies to address them. On the one hand, it is important that the person can control some of the most relevant symptoms of the illness. On the other hand, it is equally important that services promote and support users in their own recovery process, facilitating hope, or relationships with others, so that they gain autonomy to build a satisfactory life. Finally, given the role that internalized stigma has in the recovery process, it would be important to implement programs to reduce it.

## Data Availability

The datasets generated and analyzed during the current study are not publicly available since there is part of a doctoral thesis, but are available from the corresponding author on reasonable request.

## Abbreviations

SMD: Severe Mental Disorder
REE: Recovery Enhancing Environment
HoNOS: Health of the Nation Outcome Scale
GAF: Global Assessment of Functioning
CGI: Clinical Global Impression
EVA: Analogue Visual Scale
ISMI: Internalized Stigma of Mental Health Illness
CHIME: Connectedness, Hope and optimism, Identity, Meaning and purpose and Empowerment
SMDP: Severe Mental Disorder Programme
GFI: Goodness for Fit Index
AGFI: Adjusted Goodness for Fit Index
CFI: Comparative Fit Index
SRMR: Standardized Root Mean Square Residual
RMSEA: Root Mean Square Error of Approximation
